# HER2 expression and efficacy of dose-dense anthracycline-containing adjuvant chemotherapy in breast cancer patients

**DOI:** 10.1038/sj.bjc.6602660

**Published:** 2005-06-21

**Authors:** L Del Mastro, P Bruzzi, G Nicolò, G Cavazzini, A Contu, M D'Amico, A Lavarello, F Testore, B Castagneto, E Aitini, L Perdelli, C Bighin, R Rosso, Marco Venturini, and other participants

**Affiliations:** 1 Department of Medical Oncology, National Cancer Research Institute, Genoa, Italy; 2 Trials Unit, National Cancer Research Institute, Genoa, Italy; 3 Pathology Unit, National Cancer Research Institute, Genoa, Italy; 4 Ospedale C Poma, Mantova, Italy; 5 UO Oncologia Medica, ASL 1, Sassari, Italy; 6 Ospedale Galliera, Genoa, Italy; 7 Ospedale Civile, Sestri Levante (Ge), Italy; 8 Oncologia Medica, ASL 19, Asti, Italy; 9 Oncologia Medica, Ospedale Casale Monferrato, Italy

**Keywords:** erbB-2, dose density, predictive factors, prognosis

## Abstract

No data are available on the role of HER2 overexpression in predicting the efficacy of dose-dense anthracycline-containing adjuvant chemotherapy in breast cancer patients. We retrospectively evaluated this role in patients enrolled in a phase III study comparing standard FEC21 (5-fluorouracil, epirubicin, and cyclophosphamide, administered every 3 weeks) *vs* dose-dense FEC14 (the same regimen repeated every 2 weeks). HER2 status was determined for 731 of 1214 patients. Statistical analyses were performed to test for interaction between treatment and HER2 status with respect to event-free survival (EFS) and overall survival (OS); EFS and OS were compared within each HER2 subgroup and within each treatment arm. Median follow-up was 6.7 years. Among FEC21-treated patients, both EFS (HR=2.07; 95% CI 1.27–3.38) and OS (HR=2.47; 95% CI 1.34–4.57) were significantly worse in HER2 + patients than in HER2 − patients. Among FEC14-treated patients, differences in either EFS (HR=1.21; 95% CI 0.65–2.24) or OS (HR=1.85; 95% CI 0.88–3.89) between HER2 + and HER2 − patients were not statistically significant. Interaction analysis suggested that the use of dose-dense FEC14 might remove the negative prognostic effect of HER2 overexpression on EFS and OS. Our data suggest a potential role of HER-2 overexpression in predicting the efficacy of dose-dense epirubicin-containing chemotherapy and the need to confirm this hypothesis in future prospective studies.

HER-2 overexpression or amplification has been widely studied as a prognostic and predictive factor in early breast cancer patients but its role is still controversial (Trock *et al*, 2000; Ravdin, 2001; Sledge, 2001; Yamauchi *et al*, 2001; Goldhirsch *et al*, 2003). In most studies, overexpression or amplification of the HER2 gene has been associated with an adverse clinical outcome (Schnitt, 2001). With regard to HER2 as a predictive factor, retrospective studies have suggested that the benefit from anthracycline-containing chemotherapy might be greater in women whose tumour overexpresses HER2 (Paik *et al*, 1998, 2000; Di Leo *et al*, 2002). Another important issue is to evaluate if HER2 overexpression is associated not only with sensitivity to anthracyclines but also to their dose-intensity and dose-density effect. Results from a retrospective study suggest that regimens with an increased dose per cycle (dose intensity) of anthracyclines (Thor *et al*, 1998) are associated with an improved outcome only in women with HER2 overexpression. Recent data showed that anthracycline-containing chemotherapy with increased dose density (obtained by the administration of the drugs with a shortened interval), but with the same dose per cycle and total dose, improves clinical outcome as compared to conventionally scheduled (every 3 weeks) regimens (Citron *et al*, 2003; Venturini *et al*, 2003). However, no data are available on the potential role of HER2 overexpression in predicting the efficacy of dose-dense anthracycline-containing chemotherapy. The aim of this study was to evaluate the prognostic and predictive role of HER2 overexpression in early breast cancer patients enrolled in a trial that compared standard *vs* dose-dense epirubicin-containing adjuvant chemotherapy.

## PATIENTS AND METHODS

The patients evaluated in the present study were a subgroup of the study population entered into a prospective clinical trial. HER2 status was centrally evaluated in primary breast cancer samples from patients enrolled in a phase III multicentre study comparing standard *vs* dose-dense adjuvant chemotherapy (GONO-MIG-1 study, Gruppo Oncologico Nord-Ovest-Mammella Intergruppo). The GONO-MIG-1 study was conducted in 22 Italian centres enrolling 1214 patients from 1992 to 1996. Women (age ⩽70 years) with histologically confirmed breast cancer who had undergone radical mastectomy or breast-conserving surgery plus full ipsilateral axillary node dissection were eligible for enrolment in the study if they had no more than 10 involved axillary nodes or were node negative but had a high risk of recurrence. High risk was defined as the presence of one or more of the following characteristics: age ⩽35 years, negative oestrogen (ER) and progesterone receptor (PgR) status, tumour size ⩾2 cm, poor histological grade, high proliferative rate determined by [^3^H]thymidine labelling index, or by S-phase determination by flow cytometry. Patients were randomly assigned to receive either six courses of FEC21 (5-fluorouracil 600 mg m^− 2^, epirubicin 60 mg m^− 2^, and cyclophosphamide 600 mg m^− 2^ intravenously on day 1, repeated every 3 weeks) or six courses of dose-dense FEC14 (the same drugs at the same doses of FEC21, repeated every 2 weeks), with the support of filgrastim, a granulocyte colony-stimulating factor (G-CSF). Granulocyte colony-stimulating factor was subcutaneously self-administered by patients, at a dose of 5 *μ*g kg^− 1^ day^− 1^, on days 4–11. Patients with ER- and/or PgR-positive tumours received tamoxifen 20 mg day^− 1^ for 5 years. Postoperative regional radiotherapy limited to the remaining breast was given to patients who had received conservative surgery.

### Tumour sample collection

All 22 participating centres were invited to participate in this study on the role of HER2 status (HER2 study) and 18 centres accepted. Each centre was provided with a list of all patients entered into the clinical study. Each centre sent one paraffin-embedded sample of the primary tumour to the Pathology unit of the National Cancer Research Institute of Genoa, Italy. Once samples were received, they were classified and stored at room temperature until the HER2 analysis was carried out.

### HER2 evaluation

Immunohistochemical analysis was performed using monoclonal antibody CB-11 (Biogenex, San Ramon, CA, USA). Sections (3-*μ*m-thick) from formalin-fixed, paraffin-embedded tissue were cut and mounted on positively charged slides. Tissue sections were deparaffinised and rehydrated in graded alcohol. No antigen retrieval procedure was used. Slides were placed in TBS (0.05 M Tris/HCl; 0.15 M NaCl; pH 7.6) and endogenous peroxidase activity was blocked by a 5 min treatment with 3% hydrogen peroxide solution. Blocking solution was applied for 20 min, followed by incubation with the primary antibody (1 : 10 dilution TBS) for 30 min. Tissue sections received a 5 min TBS rinse before the application of biotinylate secondary antibody (1 : 200 dilution TBS) for 30 min and then a second 5 min TBS rinse. Antibody was localised using the streptavidin biotin immunoperoxidase (Dako, Dakocytomation, Milan, Italy; 1 : 10 dilution TBS), and 3′-3 diaminobenzidine was used to visualise the chromogen. Slides were counterstained in Mayer haematoxylin, dehydrated, and mounted. For each run, a composite slide of three formalin-fixed human breast carcinoma cell lines representing different levels of HER2 protein expression (MDA-231=0; MDA-175=1 +; and SKBR=3 +) was used as control. In addition, an overexpressing HER2 tissue section of breast cancer was used as positive and negative control. Negative control was made by substituting the HER2 primary antibody with normal rabbit serum. When both carcinoma *in situ* and invasive carcinoma were present in the same section, only the invasive component was scored. For the determination of HER2 protein overexpression, only the membrane staining intensity and pattern were evaluated as follows: no staining or membrane staining in less than 10% of the tumour: score 0; a faint/barely perceptible membrane staining in more than 10% of the tumour cells, stained only in part of their membrane: score 1 +; a weak to moderate complete membrane staining in more than 10% of the tumour cells: score 2 +; a strong complete membrane staining in more than 10% of tumour cells: score 3 +.

In this study, patients were considered HER2 positive (HER2 +), that is, with overexpression of HER2, if it was scored as 3 +; patients with HER2 scored as 0, 1 +, 2 + were considered HER2 negative (HER2 −). The cutoff of 3 + was chosen before inspection of clinical results in order to consider as positive only tumours with unequivocal HER2 overexpression (Zarbo and Hammond, 2003).

The same pathologist, who was blinded to both treatment assignment and clinical outcome, scored all slides.

### Statistical methods

Overall survival (OS) was estimated from the date of randomisation to the date of last contact or death from any cause. Event-free survival (EFS) was defined as the interval that had elapsed between the date of randomisation and the date of local relapse, distant relapse, second breast primary, or death from any cause, whichever occurred first.

All selected patients, regardless of eligibility or of compliance to the assigned treatment, were considered in the arm they were assigned at randomisation in the original study and there were no further exclusions.

The prognostic role of HER2 status on EFS and OS was assessed by fitting a multivariate proportional hazard model to the data. The following variables were initially included in the model: treatment assigned at randomisation, age, menopausal status, tumour size, nodal status, grading, ER status, PgR status, proliferative activity, and HER2 status. The final model was obtained by means of a step-down procedure based on the likelihood ratio test. In order to retain in the final model as many potential confounding factors as possible, it was decided, prior to data analysis, to use relaxed significance levels (i.e. *P*<0.15). The heterogeneity of the effect of the adjuvant regimen (dose dense *vs* standard) according to HER2 status was investigated by including in each final model (for EFS and OS) an interaction term representing the modification of the effect of dose-dense therapy in HER2 + patients. For descriptive purposes, OS and EFS in patients assigned to dose-dense or standard adjuvant therapy were also compared separately within each HER2 subgroup. Kaplan–Meier estimates and plots were used in all univariate analyses.

## RESULTS

Results regarding all patients (*n*=1214) enrolled in the clinical trial GONO-MIG1 have been recently presented (Venturini *et al*, 2003). At a median follow-up of 6.7 years, a 19% reduction in the hazard of death was observed in favour of the FEC14 arm.

### Collection of tumour samples

Four out of 22 centres involved in the GONO-MIG1 study, which had enrolled 164 cases, chose not to participate in the HER2 study. The 18 centres participating in the HER2 study enrolled 1050 patients in the GONO-MIG1 study.

A total of 731 paraffin-embedded samples of primary tumours suitable for HER2 analysis were collected between December 1998 and February 2000, corresponding to 60% of the overall trial population (1214 patients) and to 70% of the patients recruited in centres participating in this study (1050 patients). Reasons for the lack of HER2 evaluation in 319 cases were the following: tumour specimen not available (294 cases); tumour specimen inadequate (23 cases); unknown (two cases).

### Study population

To explore if the subgroup of patients evaluated in the HER2 study was representative of the whole population entered in the clinical trial, the main patient and tumour characteristics in this subgroup (731 cases) were compared with those of the subgroup without HER2 assessment (483 cases). No major difference was observed between the two subgroups (Table 1). Among the 731 patients evaluated in this study, no differences were observed between the two treatment arms. Tumour characteristics and chemotherapy regimen by HER2 status are shown in Table 2. HER2 was overexpressed, that is, 3 + (HER2 +), in 13.5% of FEC14-treated patients and in 14.7% of FEC21-treated patients. A negative hormone receptor status (i.e. both ER and PgR negative) was observed more frequently in tumours overexpressing HER2 (62%) than in HER2-negative (32.5%) tumours.

### HER2 overexpression as a prognostic factor

**Figure 1:**
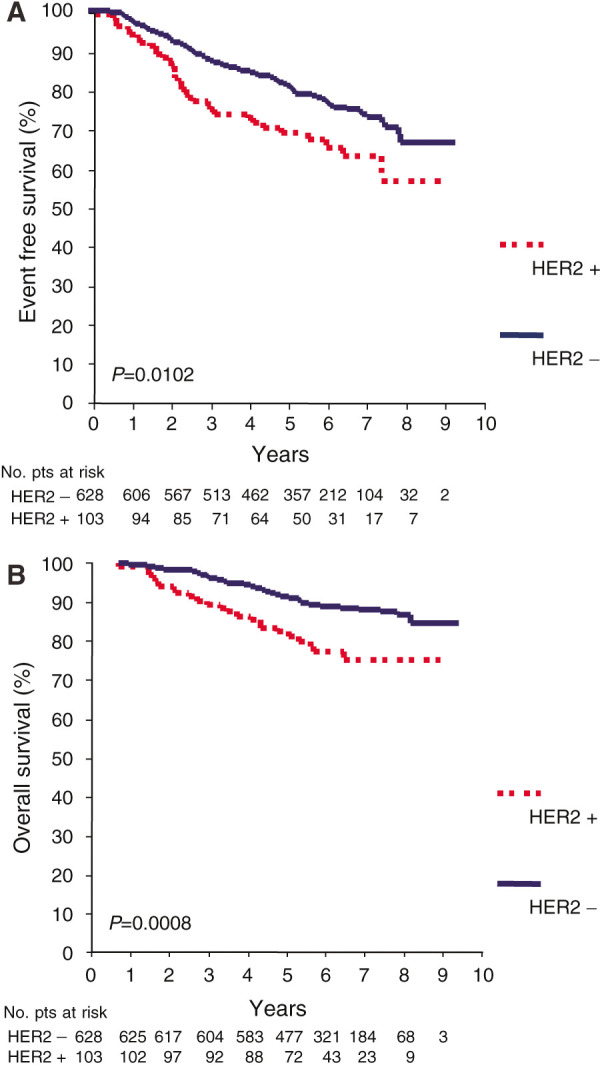
Event-free survival (**A**) and OS (**B**) curves of HER2-positive (HER2 +) *vs* HER2-negative (HER2 −) patients.

HER2 overexpression was associated with a poor prognosis. Event-free survival was significantly worse in HER2 + patients than in HER2 − patients. At a median follow-up of 6.7 years, 33 recurrences occurred among the 103 HER2 + patients, compared to 137 recurrences among the 628 HER2 − patients for an actuarial 5-year EFS of 70 and 81%, respectively (HR=1.64; 95% CI 1.12–2.40; *P*=0.01) (Figure 1A). HER2 + patients had a significantly shorter OS than HER2 − patients, with 23 deaths occurring among the 103 HER2 + patients as compared to 69 deaths among the 628 HER2 − patients for an actuarial 5-year OS of 82 and 91%, respectively (HR=2.20; 95% CI 1.37–3.53; *P*=0.001) (Figure 1B).

In multivariate analyses, HER2 overexpression was confirmed to be a factor independently associated with poor EFS (HR=1.55; 95% CI 1.05–2.28; *P*=0.027) and OS (HR=2.00; 95% CI 1.22–3.26; *P*=0.006). Other adverse prognostic factors associated with EFS and OS were treatment with FEC21 as compared to FEC14 nodal status, PgR status, proliferative activity, tumour size, and grading (Tables 3 and 4).

A contralateral breast cancer occurred in 13 cases (three cases in HER2 + patients and 10 cases in HER2 − patients), and in two of these cases, it was concurrent with loco-regional relapse. The results of disease-free survival, where the 11 cases of contralateral breast cancer as first event were excluded, closely mirrored those of EFS analyses (data not shown).

### Interaction between HER-2 overexpression and treatment

**Figure 2:**
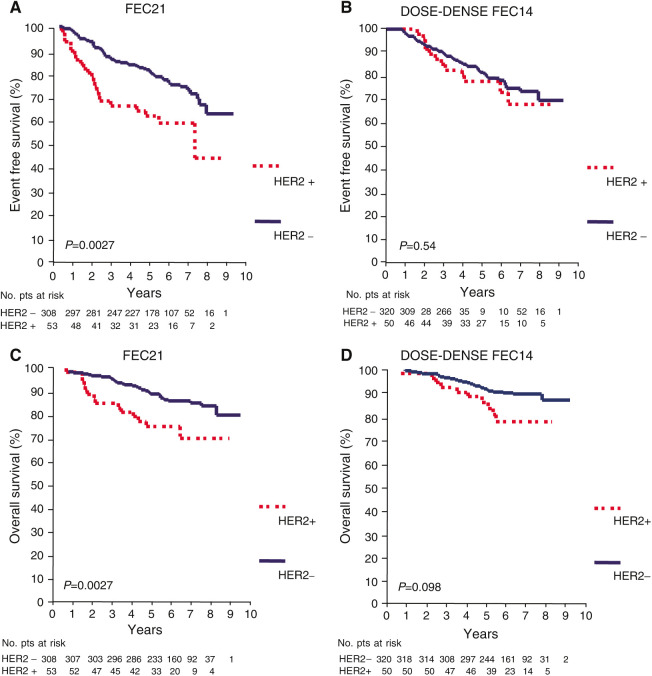
Event-free survival and OS curves of HER2-positive (HER2 +) *vs* HER2-negative (HER2 −) patients according to FEC21 (**A**–**C**) or dose-dense FEC14 (**B**–**D**) treatment.

Among patients treated with standard FEC21 regimen, both EFS (HR=2.07; 95% CI 1.27–3.38; *P*=0.003) and OS (HR=2.47; 95% CI 1.34–4.57; *P*=0.004) were significantly worse in HER2 + than in HER2 − patients (Figure 2A and C). Conversely, among patients treated with dose-dense FEC14, no statistically significant difference in either EFS (HR=1.21; 95% CI 0.65–2.24; *P*=0.54) or OS (HR=1.85; 95% CI 0.88–3.89; *P*=0.103) was observed between HER2 + and HER2 − patients (Figure 2B and D).

**Figure 3:**
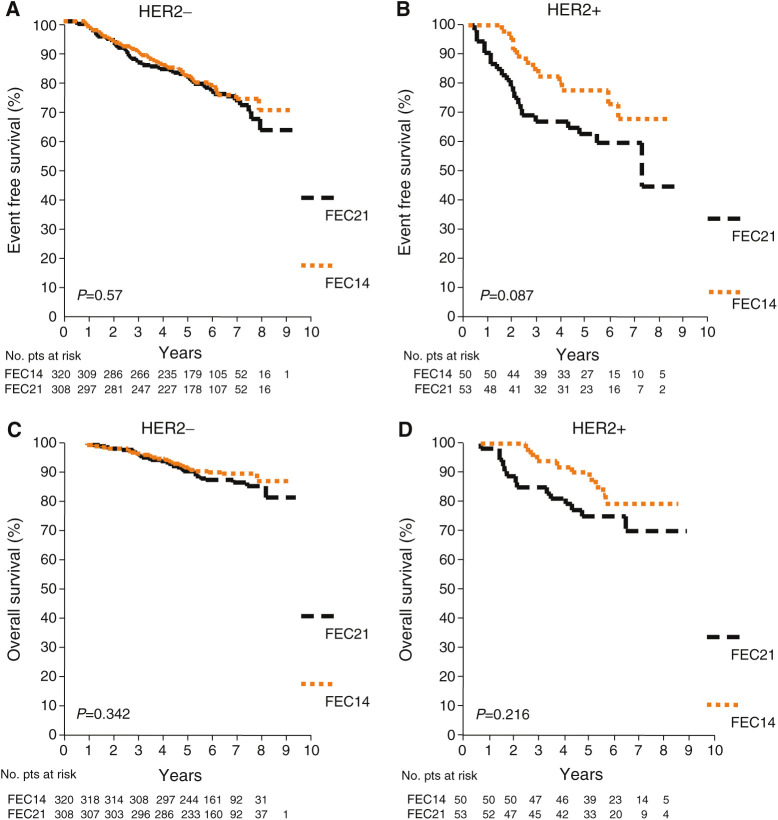
Event-free survival (**A, B**) and OS (**C**–**D**) curves of FEC21-treated patients *vs* dose-dense FEC14-treated patients according to HER2 status.

Similarly, when the outcomes of patients assigned to FEC14 were compared to those of patients assigned to FEC21, within the subgroup of HER2 − patients (*n*=628), no difference was seen in EFS (HR=0.91; 95% CI 0.65–1.27; *P*=0.57), whereas, among the smaller subgroup of HER2 + patients (*n*=103), a reduction in the rate of events was seen in patients assigned to FEC14 (HR=0.54; 95% CI 0.27–1.11; *P*=0.092), although the difference did not reach statistical significance. When analysing OS, the difference between the effect of the experimental treatment in HER2 − and HER2 + patients was less marked (HR=0.79, 95% CI 0.49–1.28, *P*=0.34 and HR=0.59, 95% CI 0.26–1.37, *P*=0.22, in HER2 − and HER2 + patients, respectively). Figure 3 shows Kaplan–Meier plots for EFS and OS. In HER2 − patients, EFS at 5 years was 80.9% (95% CI 76.3–85.5%) and 81.5% (95% CI 76.9–86.0%) in FEC21 and FEC14, respectively, and OS at 5 years was 90.7% (95% CI 87.4–94.0%) and 91.9% (95% CI 88.9–94.9%) in FEC21 and FEC14, respectively. In contrast, for HER2 + patients, Kaplan–Meier estimates of 5-year EFS were 62.5% (95% CI 49.0–75.9%) in FEC21 *vs* 77.7% (95% CI 65.5–89.9%) in FEC14 arms, and 5-year estimates of OS were 75.1% (95% CI 63.5–86.9%) in FEC21 *vs* 89.9% (95% CI 81.6–98.3%) in FEC14 arms.

When the potential modifying role of HER2 status on the effect of the experimental treatment was formally assessed by introducing the appropriate interaction term in the two multivariate models (one for EFS, the other for OS), the results were suggestive but not statistically significant for EFS (*P* for interaction=0.12), and negative for OS (*P* for interaction=0.379).

In both models, however, the estimated coefficients suggested that the use of dose-dense adjuvant chemotherapy might remove the negative prognostic effect of HER2 overexpression on EFS and partially contrasted that on OS (Table 5).

## DISCUSSION

We evaluated both the prognostic role of HER2 overexpression and its potential role as a modifier of the effect of adjuvant therapy in a population of early breast cancer patients enrolled in a phase III study comparing two epirubicin-containing regimens with different dose densities. In the overall population, regardless of the FEC regimen received (dose dense or standard), HER2 overexpression was associated with a poor prognosis. Both EFS (HR=1.64; 95% CI 1.12–2.40) and OS (HR=2.2; 95% CI 1.37–3.53) were significantly shorter in HER2 + patients than in HER2 − patients. Because all patients in our study were treated with FEC chemotherapy, our results indicate that HER2 overexpression retains its prognostic role also in patients receiving anthracycline-containing chemotherapy.

The role of HER2 in predicting sensitivity to anthracyclines is still under investigation. Clinical data suggest that women whose tumours overexpress HER2 might derive greater benefit from anthracycline-based than from alkylating agent-based adjuvant therapy (Paik *et al*, 1998, 2000; Di Leo *et al*, 2002). Another clinically important issue is whether HER2 overexpression or amplification is predictive of the benefit deriving from the use of anthracyclines administered at a dose intensity and/or dose density higher than the standard dose. Two retrospective studies addressed the issue of the potential association between HER2 and the increased dose intensity of anthracycline-containing chemotherapy. In both studies, the increase in dose intensity was obtained by increasing the single dose per cycle while intervals between cycles were not reduced, that is, the dose density was not increased (Thor *et al*, 1998; Di Leo *et al*, 2002). Results from CALGB 8541 study, in which patients were randomised to three different dose levels of CAF, suggest that the benefit from the higher dose-intensity CAF was confined to women with HER2 overexpression. However, the dose level defined as higher is what we now consider the standard dose, that is, 60 mg m^− 2^ of doxorubicin. The study from Piccart *et al* (2001) compared two regimens of epirubicin at different dose intensities (60 *vs* 100 mg m^− 2^ per cycle) with CMF (Piccart *et al*, 2001). A retrospective analysis carried out in 55% of the clinical trial population showed no difference in EFS between high dose-intensity and standard dose-intensity epirubicin in the small group of HER2-amplified patients. The results of the two above-cited studies indicate that the potential association between HER2 overexpression or amplification and anthracycline dose-intensity benefit remains to be clarified.

This study, to the best of our knowledge, is the first to investigate the potential role of HER2 in predicting the efficacy of a dose-dense epirubicin-containing adjuvant chemotherapy. In our study, patients received the same drug doses per cycle and the same total dose in the two arms, and the only difference between the two arms was the interval between cycles, that is, 2-week (dose dense) *vs* 3-week (standard) schedule. Our results suggest that dose-dense FEC14 may be superior to FEC21 in HER2 + patients only, even though differences in outcome did not reach statistical significance. In the HER2 + cohort, the relative risks of failure for FEC14-treated patients as compared to FEC21 were 0.54 (95% CI 0.27–1.11; *P*=0.092) for EFS and 0.59 (95% CI 0.26–1.37; *P*=0.22) for survival. In HER2 + patients, treatment with FEC14 was associated with 15.2% (95% CI − 3–33.4%) and 14.8% (95% CI 0.4–29.2%) absolute increase in 5-year EFS and OS, respectively, as compared to HER2 + patients receiving FEC21. Among patients treated with FEC21 regimen, both EFS and OS were shorter in HER2 + patients than in HER2 − patients, while when patients were treated with dose-dense FEC14, differences in outcome between HER2 + and HER2 − patients were not statistically significant.

The potential higher efficacy of dose-dense FEC14 treatment in HER2 + patients than in HER2 − patients may be biologically explained by data suggesting that HER2 overexpression confers a high proliferative capability to the tumour (Borg *et al*, 1991) and is associated with an amplification of topoisomerase II alpha gene, a potential marker of anthracycline sensitivity (Di Leo *et al*, 2002). Because the regrowth of cancer cells between cycles of cytoreduction is likely to be more rapid in HER2 + tumours, the more frequent administration of anthracycline-containing chemotherapy could be particularly effective in these anthracycline-sensitive tumours.

These results suggest a potential role of HER2 overexpression in predicting the efficacy of dose-dense epirubicin-containing adjuvant chemotherapy, but they must be considered with caution because of some weaknesses of the study. The first one is related to its retrospective nature. Even though no remarkable difference was seen between patients included and those not included in the present study, the possibility of a selection bias cannot be ruled out. The second limitation is due to its statistical power, and specifically to the small number of HER2-positive patients. As a matter of fact, with 170 events and 92 deaths, the study had limited power (<30%) in detecting plausible differences (HR between 1.25 and 1.33) in the risk of relapse and of death, particularly in interaction analyses. As a consequence, some of the observed differences fail to achieve statistical significance. A third possible limitation is the absence of FISH testing. When our study started, at the end of 1998, FISH testing on paraffin-embedded specimens was not widely available. Then, we performed our analysis using immunohistochemistry. At that time, CB11 was among the most commonly primary reagents used to determine HER2 status. Moreover, the hypothesis of an interaction between anthracycline dose intensity and HER2 overexpression was tested just by using CB11 (Thor *et al*, 1998).

Eventually, as expected, HER2 overexpression was inversely associated with the expression of hormone receptors (Table 2), leading to a more frequent use of endocrine therapy in HER2-negative patients than in HER2-positive patients. This different use of endocrine therapy may potentially confound the outcome. However, our results were obtained from multivariate analysis where the potential confounding effect of hormonal status and the consequent endocrine therapy was accounted for.

Owing to these limitations, our results should be considered exploratory and need to be confirmed in prospective, well-powered studies.

## Figures and Tables

**Table 1:** Patient characteristics by HER-2 availability

	**All patients**		**HER2 status**		**HER2 status**	
	**(*n*=1214)**		**Available (*n*=731)**		**Not available (*n*=483)**	
	**No.**	**%**	**No.**	**%**	**No.**	**%**
*Age (years)*						
Median (range)	54 (25–70)		54 (25–70)		54 (26–70)	
<50	470	38.7	262	35.8	208	43.1
50–59	425	35.0	253	34.7	172	35.6
>59	319	26.3	216	29.5	103	21.3
						
*Tumour size*						
pT1	598	49.2	344	47.1	254	52.6
pT2	542	44.6	338	46.2	204	42.2
pT3-4	60	5.0	39	5.3	21	4.4
Unknown	14	1.2	10	1.4	4	0.8
						
*Nodal status*						
Negative	431	35.5	275	37.6	156	32.3
Positive	783	64.5	456	62.3	327	67.7
						
*Grading*						
G1	63	5.1	44	6.0	19	3.9
G2	603	49.7	374	51.2	229	47.4
G3	405	33.4	234	32.0	171	35.4
Unknown	143	11.8	79	10.8	64	13.3
						
*ER status*						
Negative	500	41.1	318	43.5	182	38.1
Positive	628	51.8	392	53.6	236	49.4
Unknown	86	7.1	21	2.9	65	13.5
						
*PgR status*						
Negative	580	47.7	366	50.1	214	44.3
Positive	476	39.2	304	41.6	172	35.6
Unknown	158	13.1	61	8.3	97	20.1

ER=oestrogen receptor; PgR=progesterone receptor.

**Table 2:** Treatment arm and tumour characteristics by HER2 status

	**HER2=0 (*n*=542)**		**HER2=1 + (*n*=39)**		**HER2=2 + (*n*=47)**		**HER2=3 + (*n*=103)**	
	**No.**	**%**	**No.**	**%**	**No.**	**%**	**No.**	**%**
*Treatment arm*								
FEC14	277	51.1	19	48.7	24	51.1	50	48.5
FEC21	265	48.9	20	51.3	23	48.9	53	51.5
								
*Tumour size*								
pT1	261	48.2	15	38.5	25	53.2	43	41.7
pT2	246	45.4	19	48.7	17	36.2	56	54.4
pT3–4	30	5.5	3	7.7	4	8.6	2	2.0
Unknown	5	0.9	2	5.1	1	2.1	2	1.9
								
*Nodal status*								
Negative	206	38.0	16	41.0	13	27.7	40	38.8
Positive	336	62.0	23	59.0	34	72.3	63	61.2
								
*Grading*								
G1	38	7.0	2	5.1	3	6.4	1	1.0
G2	285	52.6	21	53.8	22	46.8	46	44.7
G3	161	29.7	11	28.2	16	34.0	46	44.7
Unknown	58	10.7	5	12.8	6	12.8	10	9.7
								
*ER status*								
Negative	215	39.7	12	30.8	22	46.8	69	67.0
Positive	316	58.3	26	66.7	22	46.8	28	27.2
Unknown	11	2.0	1	2.6	3	6.4	6	5.8
								
*PgR status*								
Negative	250	46.1	18	46.2	26	55.3	72	69.9
Positive	254	46.9	14	35.9	16	34.0	20	19.4
Unknown	38	7.0	7	17.9	5	10.6	11	10.7

ER=oestrogen receptor; PgR=progesterone receptor.

**Table 3:** Multivariate analysis – effect of various prognostic factors on EFS

**Factor strata**	**No. of patients**	**Events**	**HR**	**95% CI**	***P***
*Random*					
FEC21	361	92	1 (ref)		
FEC14	370	78	0.75	0.55–1.02	0.067
					
*Age (years)*					
<50	262	55	—		
50–59	253	59	—		
>59	216	56	—		0.68^a^
					
*Menopausal status*					
Pre	293	61	—		
Post	438	109	—		0.469^a^
					
*Nodal status*					
N −	275	33	1 (ref)		
N +	456	137	3.37	2.28–5.00	<0.0001
					
*Grading*					
1	44	5	—		
2	374	79	—		
3	234	66	—		
Unknown	79	20	—		0.23^a^
					
*ER status*					
Negative	318	82	—		
Positive	392	81	—		
Unknown	21	7	—		0.81^a^
					
*PgR status*					
Negative	366	101	1 (ref)		
Positive	304	57	0.58	0.41–0.81	
Unknown	61	12	0.54	0.29–0.98	0.002
					
*Proliferative activity*					
Low	189	31	1 (ref)		
High	263	69	2.11	1.36–3.25	
Unknown	279	70	1.80	1.17–2.76	0.003
					
*Tumour size*					
pT1	344	61	1 (ref)		
PT2/3/4/unknown	387	109	1.74	1.27–2.387	0.01
					
*HER2*					
Negative	628	137	1 (ref)		
Positive	103	33	1.55	1.05–2.28	0.027

EFS=event-free survival; ER=oestrogen receptor; PgR=progesterone receptor.

a Removed from the final model.

**Table 4:** Multivariate analysis – effect of various prognostic factors on OS

**Factor strata**	**No. of patients**	**Events**	**HR**	**95% CI**	***P***
*Random*					
FEC21	361	52	1 (ref)		
FEC14	370	40	0.65	0.43–0.98	0.041
					
*Age (years)*					
<50	262	27	—		
50–59	253	34	—	—	
>59	216	31	—	—	0.600^a^
					
*Menopausal status*					
Pre	293	31	—		
Post	438	61	—		0.246^a^
					
*Nodal status*					
N −	275	14	1 (ref)		
N +	456	78	4.34	2.42–7.77	<0.0001
					
*Grading*					
1	44	4	1 (ref)		
2	374	36	0.85	0.30–2.42	
3	234	39	1.30	0.45–3.77	
Unknown	79	13	1.77	0.57–5.51	0.038
					
*ER status*					
Negative	318	52	—		
Positive	392	35	—		
Unknown	21	5	—		0.68^a^
					
*PgR status*					
Negative	366	61	1 (ref)		
Positive	304	26	0.49	0.31–0.79	
Unknown	61	5	0.31	0.12–0.80	0.001
					
*Proliferative activity*					
Low	189	15	1 (ref)		
High	263	36	2.20	1.19–4.08	
Unknown	279	41	2.02	1.11–3.67	0.02
					
*Tumour size*					
PT1	344	24	1 (ref)		
PT2/3/4/unknown	387	68	2.52	1.58–4.02	<0.0001
					
*HER2*					
Negative	628	69	1 (ref)		
Positive	103	23	2.00	1.22–3.26	0.006

OS=overall survival; ER=oestrogen receptor; PgR=progesterone receptor.

a Removed from the final model.

**Table 5:** Results of interaction analyses

**Factor**	**Coefficient (s.e.)**	**HR**	***P***
*EFS* a			
Random (FEC14 *vs* FEC21)	− 0.166 (0.173)	0.85	0.335
HER2 status (positive *vs* negative)	0.713 (0.254)	2.04	0.005
Random × HER2 status	− 0.633 (0.405)	0.53	0.118
			
*OS* a			
Random (FEC14 *vs* FEC21)	− 0.328 (0.245)	0.72	0.180
HER2 status (positive *vs* negative)	0.881 (0.321)	2.41	0.006
Random × HER 2 status	− 0.438 (0.497)	0.646	0.379

EFS=event-free survival; OS=overall survival.

a Estimates obtained from a model with nodal status, PgR status, proliferative index (low, high, unknown), and pT (pT1 *vs* greater).

## Appendix A1

**Other participants**

Ospedale S Lazzaro Alba (CN), Italy: Gianfranco Porcile and Paolo De Giuli

Ospedale Civile, USL 8, Arezzo, Italy: Paolo Ghezzi and Vincenzo Sforza

ASL 19, Asti, Italy: Elda Feyles

Ospedale Oncologico Businco, Cagliari, Italy: Vittorio Mascia, Efisio De Fraia, and Antonello Ferreli

Ospedale Santo Spirito, Casale Monferrato, Italy: Mansueto Pavesi

Policlinico Careggi, Firenze, Italy: Paolo Pacini and Giuseppe Zampa

Ospedali Galliera, Genova, Italy: Roberto Bandelloni

Istituto Nazionale per la Ricerca sul Cancro, Genova, Italy: Francesco Boccardo

Ospedale S Andrea, La Spezia, Italy: Paolo Pronzato and Franco Fedeli

Ospedale C Poma, Mantova, Italy: Alberto Bellomi

Ospedale S Leopoldo, Mandic di Merate, Italy: Stefano Banducci and Mancosu

Ospedale S Raffaele, Milano, Italy: Enrico Villa and Angelo Cantaboni

Presidio Ospedaliero, S Remo, Italy: Elisabetta Campora and Corrado Ruggieri

Az. USL N1 – Ospedale SS Annunziata, Sassari, Italy: Antonio Cossu

Ospedale S Paolo, Savona, Italy: Fulvio Brema and Cristina Quaglia

Ospedale Civile, Sestri Levante, Italy: Rubizzo

Azienda Ospedaliera Triestina, Trieste, Italy: Giorgio Mustacchi and Furio Silvestri
